# A new species of genus *Tetrasticta* Kraatz (Coleoptera, Staphylinidae, Aleocharinae) from Xishuangbanna, Southwest China

**DOI:** 10.3897/zookeys.404.7276

**Published:** 2014-04-24

**Authors:** Dan-Lin Zheng, Mei-Jun Zhao

**Affiliations:** 1 Department of Biology, College of Life and Environmental Sciences, Shanghai Normal University, 100 Guilin Road, Xuhui District, Shanghai 200234, P. R. China

**Keywords:** Aleocharini, first record, *Tetrasticta*, Yunnan, Xishuangbanna

## Abstract

*Tetrasticta bobbii* Zheng & Zhao, **sp. n.**, collected in Nangongshan, Xishuangbanna, Yunnan, is described and illustrated.

## Introduction

The aleocharine genus *Tetrasticta* Kraatz, 1857 (Aleocharini) contains 13 species worldwide (Maruyama and Sugaya 2002; Maruyama 2004; Pace 2000, 2008, 2013b; Yamamoto and Maruyama 2013). Pace (2010) synonymized *Creochara* Cameron, 1931 with *Tetrasticta* and repeated this arrangement in his recent paper (Pace 2013a). According to Yamamoto and Maruyama (2013), the synonymization of *Tetrasticta* by Pace (2010) does not provide sufficient evidence and should not be consider as valid. Currently, no species of this genus has been reported from Mainland China. In 2003, our colleagues Jia-Yao Hu ad Liang Tang surveyed the staphylinid fauna of Nangongshan (Yunnan, Southwest China), and collected a small series of *Tetrasticta* specimens. A closer examination of this material revealed that the species was undescribed.

## Material and methods

All the types are deposited in the Insect Collection of Shanghai Normal University, Shanghai, China (SNUC).

Specimens were killed with ethyl acetate and preserved in 75% ethanol before dissection; photos of habitus were taken with a Canon EOS 7D with an MP-E 65mm macro photo lens.

The following abbreviations are applied in the text: BL – body length, from the anterior margin of the head to the posterior margin of the abdominal tergite VIII; FBL – forebody length, from the clypeal anterior margin to the posterior margin of elytra; HD – head length, from the clypeal anterior margin to the occipital constriction; PL – length of the pronotum along the midline; HW – width of the head across the eyes; PW – maximum width of the pronotum.

## Taxonomy

### Tetrasticta bobbii sp. n.

http://zoobank.org/04ED9579-704E-4F46-9769-BD73955B5A4B

http://species-id.net/wiki/Tetrasticta_bobbii

Fig. 1

#### Type material.

**Holotype:** **China:** ♂, labelled ‘CHINA: Yunnan Prov., Xishuangbanna, Mengla County (勐腊县), Nangongshan (南贡山), alt. 800–1000 m, 7.VII.2003, Hu & Tang leg. / HOLOTYPE [red], *Tetrasticta bobbii* sp. n., Zheng & Zhao det. 2014, SNUC’. **Paratypes: China:** 1 ♂, 1 ♀ (preserved in a small tube filled with 75% ethanol), same data as holotype, both bearing the following label: ‘PARATYPE [yellow], *Tetrasticta bobbii* sp. n., Zheng & Zhao det. 2014, SNUC’.

#### Description.

**Figure 1.**
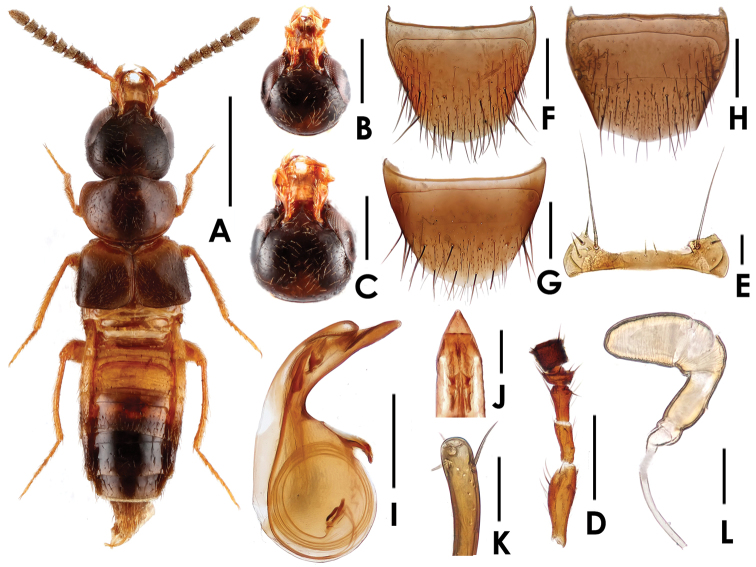
*Tetrasticta bobbii* sp. n. **A** male habitus, in dorsal view **B** female head, in dorsal view **C** male head, in dorsal view **D** antennomere I–V **E** mentum **F** male tergite VIII **G** male sternite VIII **H** female tergite VIII **I** median lobe of aedeagus, in lateral view **J** ditto, apical part, in ventral view **K** apical lobe of paramerite, in lateral view **L** spermatheca. Scales (mm): **A** = 1; **B, C** = 0.5; **D** = 0.3; **E** = 0.05; **F, G, H, I** = 0.2; **J, K, L** = 0.05.

Body (Fig. 1A) shining. Coloration: head black; antennae and pronotum reddish brown; elytra reddish brown with anterior margin reddish yellow; legs reddish yellow; abdomen with tergites II–IV reddish yellow, tergites VI–VII black.

Head (Figs 1B, C) almost 1.05 times as wide as long; slightly narrower than pronotum; surface sparsely covered with yellow setae; eyes large. Antennae (Figs 1A, D) with segment I long, as long as combined length of segments II–III; segments II and III about one-half of segment I; segment IV extremely short, much wider than long; segments IV–V almost as wide as long; segments VI–X wider than long. Mandibles long, slender. Mentum distinctly transverse, about 3.62 times as wide as long; shaped as in Fig. 1E. Pronotum wider than long, about 1.38 times as wide as long; surface moderately covered with yellow setae; disc with three shallow depressions; shaped as in Fig. 1A. Elytra wider than long; surface moderately covered with yellow setae. Abdomen flattened, with subparallel lateral margins, widest at segments IV–V; tergite VIII with six pairs of macrosetae; sternite VIII (Fig. 1G) generalized in shape, posterior margin convex in the middle, with eight pairs of macrosetae.

Male: postocular margins straight for a short distance and then narrowed posteriorly (Fig. 1C); posterior margin of tergite VIII (Fig. 1F) broadly convex; median lobe of aedeagus (Figs 1I, J )slightly narrowed apically in lateral view; inner sac with flagellum coiled five times; apical lobe of paramerite (Fig. 1K) slightly dilated, apically with four setae.

Female: postocular margins immediately narrowed behind eyes (Fig. 1B); tergite VIII shaped as in Fig. 1H; spermatheca shaped as in Fig. 1L.

#### Distribution.

Southwest China: Yunnan.

#### Measurements.

**Male:** BL: 3.81–4.00; HL: 0.79–0.82; HW: 0.83–0.85; PL: 0.64–0.65; PW: 0.88–0.90; HW/HL: 1.05–1.06; PW/PL: 1.37–1.38; HW/PW: 0.94–0.95. **Female:** BL: 3.62; HL: 0.70; HW: 0.74; PL: 0.61; PW: 0.85; HW/HL: 1.06; PW/PL: 1.40; HW/PW: 0.87.

#### Remarks.

*Tetrasticta bobbii* is most similar to *Tetrasticta gnatha* in overall body shape, relatively long mandibles, but can be readily distinguished form it by the distinctly long antennal segments II–III, the different shape of abdominal tergite VIII and the form of aedeagal median lobe.

#### Etymology.

Named after the Pomeranian dog of senior author.

## Supplementary Material

XML Treatment for Tetrasticta bobbii
